# Biomarkers for Immune Checkpoint Inhibitors in Melanoma

**DOI:** 10.3389/fonc.2018.00270

**Published:** 2018-07-18

**Authors:** Shigehisa Kitano, Takayuki Nakayama, Makiko Yamashita

**Affiliations:** ^1^Department of Experimental Therapeutics, National Cancer Center Hospital, Tokyo, Japan; ^2^Division of Cancer Immunotherapy, Exploratory Oncology Research and Clinical Trial Center, National Cancer Center, Tokyo, Japan

**Keywords:** biomarker, immune checkpoint inhibitor, malignant melanoma, cytotoxic T-lymphocyte-associated antigen 4, programmed death-1

## Abstract

Immune checkpoint inhibitors have now become a standard therapy for malignant melanoma. However, as immunotherapies are effective in only a limited number of patients, biomarker development remains one of the most important clinical challenges. Biomarkers predicting clinical benefit facilitate appropriate selection of individualized treatments for patients and maximize clinical benefits. Many biomarkers derived from tumors and peripheral blood components have recently been reported, mainly in retrospective settings. This review summarizes the recent findings of biomarker studies for predicting the clinical benefits of immunotherapies in melanoma patients. Taking into account the complex interactions between the immune system and various cancers, it would be difficult for only one biomarker to predict clinical benefits in all patients. Many efforts to discover candidate biomarkers are currently ongoing. In the future, verification, by means of a prospective study, may allow some of these candidates to be combined into a scoring system based on bioinformatics technology.

## Introduction

In recent years, immune checkpoint inhibitors have increasingly been applied to the clinical development of cancer immunotherapy. For malignant melanoma, ipilimumab, a humanized monoclonal antibody (mAb) that blocks cytotoxic T-lymphocyte-associated antigen 4 (CTLA-4) and nivolumab, as well as pembrolizumab, a humanized mAb that blocks programmed death-1 (PD-1) on primed T cells, have been approved and are now used as standard therapies. Several clinical trials have investigated new agents, alone and in combination, for use in the treatment of advanced malignant melanoma. However, immunotherapies are effective in only a limited number of patients and severe immune-related adverse events (irAEs) develop in some patients. Biomarkers predicting clinical benefit support appropriate the selection of individualized treatments for patients and maximize clinical benefits. Thus, one of the most important tasks for advancing this form of therapy is to identify “baseline (pretreatment)” biomarkers predicting responses or toxicities. In general, biomarkers are mainly divided into two functional categories, “prognostic” and “predictive.” A prognostic biomarker can be defined based on the effects of patient or tumor biology on the patient’s clinical outcome. This includes patients at high risk for disease relapse who may thus derive benefit from earlier treatments. On the other hand, a predictive biomarker is defined by the effects of treatment, including tumor response and improvements in overall survival (OS), disease-free survival (DFS), and progression-free survival (PFS). Many biomarker candidates have been identified, to date, in retrospective settings. This review summarizes recent findings of biomarker studies designed to identify means of predicting the clinical benefits of immunotherapies in melanoma patients, focusing on three categories: tumor tissue, peripheral blood, and others (Table 1).

**Table 1:** Biomarkers for metastatic melanoma patients treated with immune checkpoint inhibitor therapy.

Tumor (microenvironment)	Reference
Immunohistochemistry (IHC)	
Programmed death-ligand 1 (PD-L1) expression on tumor cells	(1–4)
PD-L1 expression on immune cells	(5–7)
Programmed death-1 expression on T cells	(25)
Infiltration of CD8^+^ cells	(37–41)
Infiltration of CD4^+^ cells	(40)
Regulatory T cells (Tregs)	(29, 30)
Myeloid-derived suppressor cells (MDSC)	(31–35)
Tumor-associated macrophages (M2)	(36)
Gene profiling (expression/mutation/amplification)	
Tumor mutation burden	(9–14)
Number of somatic mutations (non-synonymous mutations)	(10, 11, 15–19)
Activation of IFN-γ signaling	(21, 22)
Amplification of WNT/β-catenine signaling	(23)
Janus kinase (JAK) 1/JAK2 loss-of-function mutations	(25, 26)

**Peripheral blood**	

Number of lymphocytes	(42, 43)
Number of Tregs	(38, 44, 45)
Number of MDSCs	(46–50)
Number of proliferating CD8^+^ T cells	(52–57)
Number of memory CD4^+^ T cells	(58–60)
Concentrations of cytokines (e.g., IL-6, IL-8, IL-10, and TGF-β)	(62–64)
Concentration of VEGF	(66)
PD-L1 expression on circulating tumor cells	(8)
Soluble PD-L1	(67)

**Others**	

Microbiome	(68–70)
Fatty acids	(71)
Vitiligo and rash	(72–75)

## Biomarkers in Tumor Tissue

### PD-L1 Expression on Tumor Cells

Programmed death-ligand 1 (PD-L1) expression has been investigated as a potential biomarker for PD-1 or the PD-L1 inhibitor. In phase I trials, PD-L1 expression on tumor cells correlated with the response to anti-PD-1 antibody (1). Given these promising results, several companies developed PD-L1 companion diagnostic tests for anti-PD-1/PD-L1 antibody and patients with PD-L1-positive tumors were considered to be good candidates for anti-PD-1/PD-L1 antibody treatment. In fact, the U.S. Food and Drug Administration has approved pembrolizumab, an anti-PD-1 antibody, for the treatment of PD-L1-positive non-small cell lung cancer (NSCLC) and gastric cancer. However, there are several problems while using PD-L1 expression as a biomarker for immunotherapy. First, PD-L1 expression levels show heterogeneity within tumors (2). Second, PD-L1 is a dynamic marker that can be affected by treatment and local inflammation (3). Third, the optimal threshold level of PD-L1 expression remains uncertain (4). In fact, some PD-L1 negative patients also derive benefit from treatment with an anti-PD-1/PD-L1 inhibitor.

Interestingly, PD-L1 expression on tumor infiltrating immune cells may be more predictive of responsiveness to anti-PD-1 antibody than the level of PD-L1 expression by the tumor (5). Furthermore, while PD-L1 expression on tumor cells did not tend to be related to the response rate in melanoma patients treated with anti-PD-1 antibody (nivolumab) and anti-CTLA-4-antibody (ipilimumab), there was a correlation with a good response in non-small lung cancer patients treated with these drugs (6, 7). On the other hand, Schott et al. reported that PD-L1 expression on “circulating” tumor cells might also be a potential biomarker (8). They suggested circulating tumor cells to possibly be precursors of metastatic disease, with PD-L1 expression allowing stratification according to the anticipated response to therapy. Further study is needed to determine the clinical significance of PD-LI expression.

### Genes: Mutation-Burden and Gene-Expression

Melanoma is characterized by having one of the highest mutation burdens of any cancer (9, 10). These somatic mutations generate immunogenic-neoantigens recognized as tumor-antigens, possibly triggering effective anti-tumor immune responses (11–13). Genomic analysis revealed that a high mutational load at baseline may predict better survival but not treatment responses (13), and the mutation burden after PD-1 therapy was reportedly decreased in melanoma patients who responded to treatment (14).

Genes harboring significant mutations included *BRAF, CDKN2A, NRAS, PTEN*, and *TP53* in cutaneous melanoma, *BRAF, NRAS, NF1*, and *KIT* in acral melanoma (hands and feet), and *SF3B1* in mucosal melanoma (internal body surfaces) (15–17). The *BRAF* mutation was the most common, being detected in approximately half of metastatic melanoma patients. In the current treatment of melanoma, only *BRAF* V600 mutations are regarded as being molecular markers applicable to treatment decision-making strategies (10, 18). Several studies of CTLA-4 and PD-1 therapy have revealed that *BRAF* V600E mutations do not correlate with either the response to CTLA-4 therapy or the resulting OS, whereas the correlation with the response of melanomas to PD-1 therapy was significant (11, 19). On the other hand, inactivation of *CDKN2A* and/or *PTEN* is regarded as an important mechanism underlying resistance and/or durable responses to BRAF-inhibitor-based therapy, but is not currently taken into consideration in the clinical decision-making process (10).

Previous sequence studies, such as The Cancer Genome Atlas study, used exome and low-pass whole-genome sequencing (WGS). In 2017, Hayward et al. reported the first large, high-coverage WGS study of melanomas (cutaneous, acral, and mucosal subtypes), including analysis of the non-coding region. Their report showed that the number of mutations in the non-coding region was detected as a number equivalent to that in the coding region, and that the most common mutations in the non-coding region were in the *TERT* promoter upstream from the initiation codon (69% of all melanomas and 86% of cutaneous melanomas) (17). Moreover, Ishida et al. preliminarily reported a correlation between HLA-A*26 alleles and the response to anti-PD-1 (nivolumab) therapy in Japanese patients with metastatic melanoma (20). HLA accounts for some of the individual differences in antigen-specific immune responses, and might provide useful information for devising individualized immunotherapeutic regimens. The associations of these new findings with clinical responses to immunotherapies merit further investigation.

On the other hand, there have been several investigations of the gene expressions on tumor tissues, for their value in predicting responses to immune checkpoint inhibitors. Immunohistochemistry and gene profiling assays have suggested the presence of a “T-cell-inflamed tumor microenvironment,” with an abundance of chemokines and an IFN-γ signature, to correlate with the clinical efficacy of immune checkpoint inhibitors in melanoma patients (21, 22). Numerous studies have revealed the molecular mechanisms underlying lack of T-cell infiltration and resistance of melanomas to immune checkpoint therapy, such as the melanoma-intrinsic active WNT/β-catenin-signaling pathway (23) and enrichment for mutations in *PTEN* (24), loss-of-function mutations in Janus kinase (JAK1)/JAK2 (which are involved in IFNγ signaling), and β2 microglobulin (an MHC class I subunit) (25, 26).

### Tumor Infiltrating Lymphocytes (TILs)

Tumor infiltrating lymphocytes, such as T cells, macrophages, and various types of immune suppressive cells, are considered to be the most important players in the regulation of anti-tumor immune responses. Several studies have demonstrated an increase in the TIL number to correlate with good clinical responses and a higher survival rate of patients with melanoma and various other cancers (27, 28).

In melanoma patients, immune suppressive cells, such as regulatory T cells (Tregs) (29, 30), monocytic myeloid-derived suppressor cells (m-MDSCs) (31–35), and tumor-associated (activated) macrophages (TAM; M2) (36), were reportedly increased in number and thereby inhibited effector T cells, resulting in an increase in tumor growth.

In contrast, a number of investigators have reported the quantity of infiltrating CD8^+^CD45RO^+^ effector memory T cells to be clearly associated with longer DFS and OS, for many cancer types including melanoma (37–39). Recently, Wei et al. comprehensively profiled the effects of CTLA-4/PD-1-targeted immunotherapy on tumor infiltrating immune cells. Their study revealed that PD-1 blockade and CTLA-4 blockade both led to a subset of exhausted-like CD8^+^ T cells (CD45RO^+^PD-1^+^T-bet^+^EOMES^+^). They also showed that CTLA-4 blockade induced the expansion of an ICOS^+^ Th1-like CD4 effector population (CD45RO^+^PD-1^lo^TBET^+^ and CD69^+^) in melanoma patients. These observations suggested that these two immunotherapies target specific subsets of exhausted-like CD8^+^ T cells, but drive different cellular mechanisms to induce tumor rejection (40). Moreover, Canale et al. described high expression of CD39 on CD8^+^ infiltrating T cells as being increased in melanoma lesions. CD39 is the immunosuppressive enzyme termed ATP ectonucleotidase, and CD39^high^CD8^+^ T cells reportedly exhibit features of cellular exhaustion, such as reduced production of tumor necrosis factor and interleukin (IL)-2, as well as expressions of co-inhibitory receptors (41).

## Biomarkers in Periferal Blood

### Peripheral Blood Mononuclear Cells (PBMCs)

Blood biomarkers have most frequently been analyzed for correlations with clinical responses to immunotherapies. Baseline and/or post-treatment changes in absolute counts of white blood cells, lymphocytes, eosinophils, neutrophils, and monocytes, as well as ratios of neutrophils or monocytes to lymphocytes may both be promising and routinely available blood markers that have shown associations with responses to immune checkpoint inhibitors (11, 42, 43).

Recently, several studies have raised the possibility of circulating immune cells as predictive biomarkers for immune checkpoint inhibitors. The frequency of circulating Tregs is reportedly associated with disease progression and poor patient survival for many carcinomas treated with immunotherapy (38, 44, 45). Numerous studies have found that high levels of circulating m-MDSCs in various forms of cancer, including melanoma, correlate with poor survival (46–48). In patients treated with anti-PD-1 antibody, m-MDSCs were reported to be a blood cytology marker showing significant correlations with all outcome parameters (49, 50). However, human MDSCs have yet to be clearly characterized both biologically and phenotypically. A very recent study demonstrated that the frequency of CD14^+^CD16^-^HLA-DR^hi^ monocytes predicts both PFS and OS of melanoma patients treated with anti-PD-1 antibody, based on analysis employing high-dimensional single-cell mass cytometry (51). This CD14^+^ population including MDSCs might be useful as a predictive and/or prognostic biomarker for cancer patients receiving immunotherapy, but further investigation is needed to clarify the phenotype and biological characteristics of this diverse population of cells.

On the other hand, several studies examining circulating T cells have shown the involvement of CD8^+^ T cells, such as the proliferating (Ki67^+^) CD8^+^ effector-like T cells, in NSCLC patients receiving PD-1-therapy (52), and neoantigen-specific circulating CD8^+^ T cells in melanoma (53, 54). The latter are CD8^+^ T cells expressing PD-1. In addition, two complementary reports showed that CD28, a member of the same family as PD-1 (including CTLA-4 and ICOS), expressed on CD8^+^ T cells is a key molecule in PD-1-targeted therapy (55). Hui et al. showed that “CD28 is the primary target of PD-1 signaling,” using a cell-free membrane reconstitution system. Their report revealed that PD-1 was phosphorylated in response to PD-L1 ligation, thereby preferentially inducing dephosphorylation of CD28 (but not the T cell receptor), resulting in the inhibition of T cell proliferation (56). On the other hand, Kamphorst et al. found that, in lung cancer patients, proliferating Ki67^+^PD-1^+^CD8^+^ T cells were increased in peripheral blood, and subsequently activated (CD38^+^, HLA-DR^+^) and mostly expressed CD28 (57), implying that CD28 signaling is associated with rescue of the exhausted CD8^+^ T cells in PD-1 targeted therapies. These findings are reasonable and it is interesting that CD28, belonging to the same family as PD-1, is a key molecule in PD-1-targeted therapy, although its applicability as a predictive/prognostic biomarker in melanoma patients is as yet unclear. Moreover, whether other family members, including CTLA-4 and ICOS, have similar features in immune checkpoint therapy, remains unknown. Elucidating these issues might reveal novel useful biomarkers for use alone and/or in combination with PD-1-targeted therapy. Another interesting, and potentially important, finding of these studies is that proliferating CD8^+^ effector-like T cells were reportedly increased following PD-1-targeted therapy.

Several recent studies, focusing on circulating CD4^+^ T cells, found that increases in central memory CD4^+^ T cells (CD27^+^, FAS^−^, CD45RA^−^, and CCR7^+^) (58), and IL-9-producing CD4^+^ T helper (Th9) cells (59), correlated with good clinical responses of melanoma patients to anti-PD-1 therapy. Moreover, in lung cancer patients treated with nivolumab, the frequencies of CD62L^low^CD4^+^ T cells and Tregs (CD25^+^Foxp3^+^CD4^+^) in pretreatment PBMC were reported to correlate significantly with clinical responses (60). Their ASCO presentation outlined the major differences in pre-existing immunity, among patients showing a partial response, stable disease, or progressive disease, in response to anti-PD-1 Ab, as reflected by the status of CD4^+^ T cells, i.e., the balance between primed effector and Tregs. These recent reports raised the possibility that, in peripheral blood, not only T cell exhaustion but also activation of effector CD8^+^ T cells and increases in memory T cells appear to be highly important, and not only phenotyping markers but also functional molecules can serve important roles as prognostic and/or predictive factors for immune checkpoint inhibitors. Although peripheral blood analysis may provide valuable insights into the responses of cancer patients to immune checkpoint inhibitors, more investigation is needed before these biomarkers can be applied in clinical settings.

## Others

### Soluble Factors (Serum/Circulating Factors)

Lactate dehydrogenase was frequently investigated in previous studies and showed significant correlations with OS and PFS, whereas there were no correlations with responses to treatments (61). Recently, several studies have revealed that serum cytokine levels to correlate with responses to immune checkpoint inhibitors. Sanmamed et al. showed serum IL-8 levels to be highly correlated with tumor burden changes in metastatic melanoma and NSCLC patients during treatment with anti-PD-1/anti-CTLA-4 therapy (62, 63), and Yamazaki et al. reported that pretreatment serum IFN-γ, IL-6, and IL-10 levels were significantly higher in those with tumor progression among patients with advanced melanoma given nivolumab (64). In addition, in patients with metastatic melanoma receiving nivolumab, the activity of soluble CD73, which is an enzyme that hydrolyzes extracellular AMP to adenosine, in blood was shown to be significantly associated with clinical outcomes (65). Moreover, Frankhauser et al., studying metastatic melanoma patients, reported gene expression of vascular endothelial growth factor-C (VEGF-C) to correlate markedly with both CCL21 and T cell inflammation, and that serum VEGF-C concentrations were associated with both T cell activation/expansion and clinical responses to checkpoint blockade (66).

### Soluble PD-L1 (sPD-L1)

Pretreatment sPD-L1 levels reportedly correlate with progression of advanced melanoma treated with anti-CTLA-4 or anti-PD-1 antibody. Although changes in circulating sPD-L1 in the early phase after starting treatment did not distinguish responders from non-responders, patients who had increased circulating sPD-L1 after 5 months of treatment tended to show partial responses (67). The biology of sPD-L1 remains unclear and merits further research.

### Microbiome

A vast number of microbes colonize the human body. This colonization is associated with many diseases, including various malignancies. During the past decade, the advent of metagenomic sequencing that combines next-generation DNA sequencing technologies with computational analyses has allowed us to analyze the relationships between the microbiome and various cancers. Recent studies have suggested that the gut microbiome may affect the efficacy of immune checkpoint inhibitors and, consequently, that changing the gut microbiome of a mouse or even a human patient might make tumors more responsive to immune checkpoint inhibitors. This possibility was first evaluated using preclinical models. Vétizou et al. showed that the efficacy of anti-CTLA-4 therapy was diminished in a germ-free mouse model. In addition, the use of broad-spectrum antibiotics to eliminate gut microbiota altered the anti-tumor effect of anti-CTLA-4 therapy (68). Sivan et al. reported that *Bifidobacterium* counts decreased in parallel with the anti-tumor effects of anti-PD-L1 therapy in a mouse model (69). Furthermore, Gopalakrishnan et al. indicated that anti-PD-1 immunotherapy in melanoma patients may be modulated by the gut microbiome. These researchers reported significantly higher alpha diversity and a relative abundance of Ruminococcaceae bacteria in the gut microbiome of responders (70). These findings indicated that specific organisms comprising the gut microbiome enhanced anti-tumor responses in patients treated with immune checkpoint inhibitors. Although the gut microbiome is a potential predictive marker of immunotherapy, a larger prospective study is needed to confirm these results.

### Fatty Acids

Kim et al. investigated cellular metabolome and lipidome alterations related to melanoma metastasis. Their analysis showed a progressive increase in phosphatidylinositol species with saturated and monounsaturated fatty acyl chains, as the metastatic potential of the melanoma cells rose, highlighting these lipids as possible biomarkers (71).

### Vitiligo and Rash

Immune checkpoint inhibitors have a rather unique adverse event profile, generally described as irAEs, which are most commonly observed in the skin, the gastrointestinal tract, the lungs, the liver, endocrine system, and other organs. Cutaneous irAEs are much more common adverse events in patients with melanoma than in those with other solid tumors. Although vitiligo is attributed to an autoantibody to melanocytes, the etiology of vitiligo is not understood in detail. Vitiligo occurrence has long been speculated to be related to tumor shrinkage in melanoma patients (72). Vitiligo develops in 13–26% of patients treated with nivolumab (73, 74), though grade III/IV disease is rare. Recent studies have shown vitiligo and rash to be associated with a significant OS improvement in metastatic melanoma patients treated with immune checkpoint inhibitors (73–75). Furthermore, Nakamura et al. suggested that the occurrence of vitiligo might not be regarded as an early marker of good clinical response because the mean time to vitiligo occurrence was approximately 5 months after starting nivolumab (73). The onset times of vitiligo vary considerably depending on the type of drug administered and patient features. Thus, when we use cutaneous irAEs as a biomarker for immune checkpoint inhibitors, we should take into consideration the characteristics of each drug.

## Conclusion

**Figure 1:**
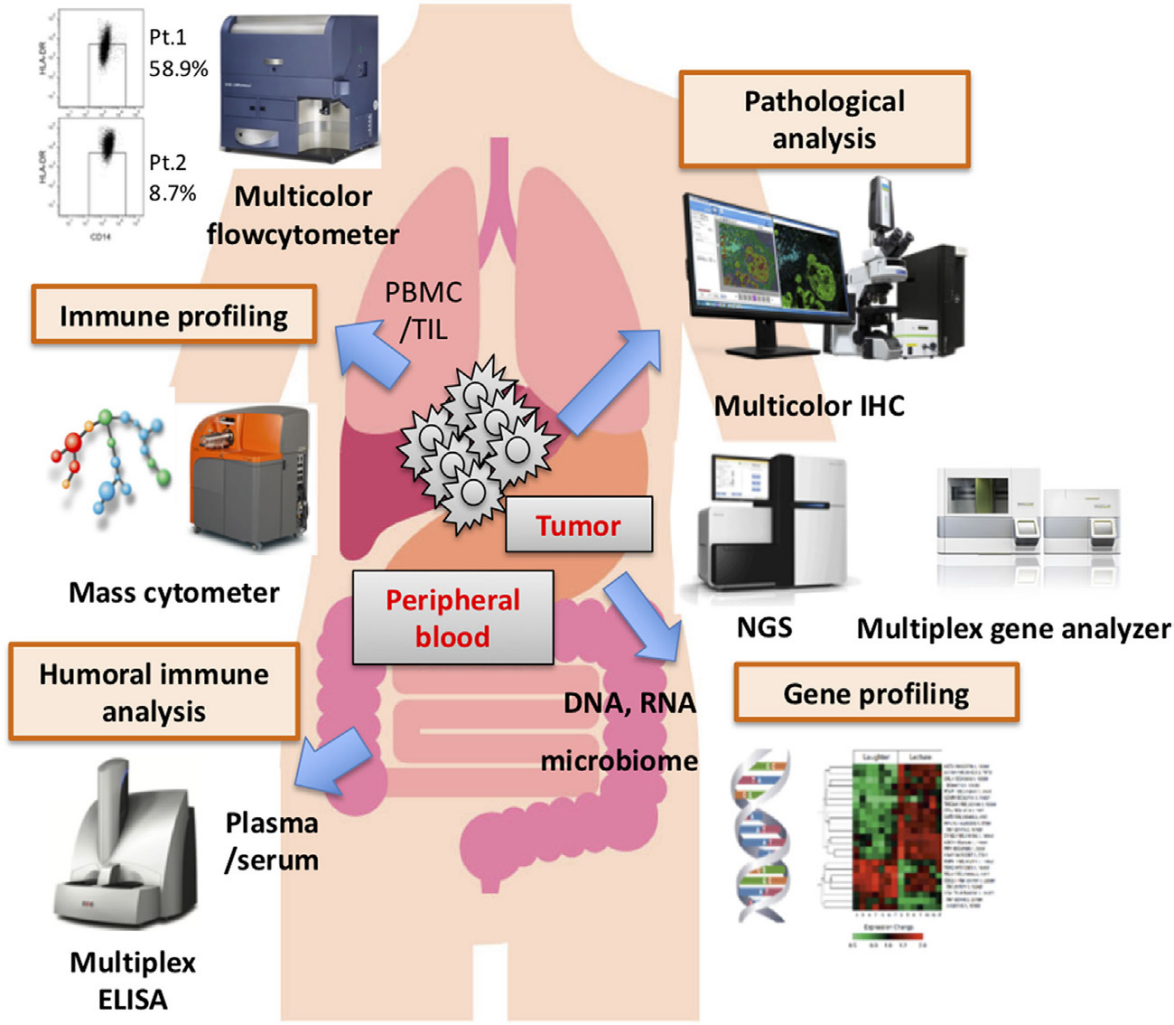
Various assay systems for identifying biomarkers. Several biomarkers derived from the tumor microenvironment, peripheral blood biology, and other factors have been proposed as distinct biomarkers of responses to immune checkpoint blockade therapy. Recently, there have been innovative advancements in assay technology that have made it possible to comprehensively profile the biology and phenotype of the tumor-microenvironment, peripheral blood, and other factors. It would be very difficult, however, for a single biomarker to predict clinical responses and/or serve as a patient selection criterion, though multifactorial biomarkers including these and other novel findings might have great value for predicting clinical responses and/or patient prognosis.

Numerous candidate biomarkers are currently the focus of research, based mainly on retrospective analyses. Most notably, tumor mutation burden, intratumoral or immune cell expressions of PD-L1, and CD8^+^ T cell infiltration into the tumor have been documented in several cohorts. For example, not only melanoma but also lung carcinoma, one of the carcinomas which also has a high mutation burden, shows good clinical responses to PD-1/PD-L1 therapy. In lung carcinoma, mutation burden, TIL accumulation, and/or PD-L1 expression on tumor cells correlated with good clinical responses. However, renal cell carcinoma is also reportedly responsive to PD-1 therapy, despite having a low mutation burden, while TIL accumulation and PD-L1 expression did not correlate with treatment effectiveness. These observations suggest that these factors are not always applicable to predicting clinical benefits. Taking into account the complex interactions between the immune system and malignancies *via* cell surface molecules, such as immune checkpoint molecules, humoral factors, including proteins, cytokines, and so on, it is not unreasonable to speculate that a single biomarker would not allow clinical benefits to be predicted in all patients. In the near future, by applying bioinformatics technology, several biomarkers might be combined to produce a useful scoring system, depending on the type of cancer, the stage, individual treatments, and the timing of intervention. Recent advancements in assay technology, such as mass cytometry (CyTOF), multicolor IHC, multiplex gene analyzer, and so on (Figure 1), have the potential to provide an abundance of biological and/or phenotypical observations in a range of environments. Now is the time to discover the candidate biomarkers which might comprise such a future scoring system. Finally, needless to say, a prospective study on a large patient population is essential.

## Author Contributions

SK: conception/design of the manuscript. SK, TN, and MY: writing of the manuscript.

## Conflict of Interest Statement

The authors have no conflicts of interest to disclose. Outside of the submitted work, SK reports personal fees from Astra Zeneca, personal fees from Chugai, personal fees from Pfizer, personal fees from Sanofi, personal fees from Nippon Kayaku, personal fees from Boehringer Ingelheim, personal fees from Meiji Seika Pharma, personal fees from Taiho, personal fees from Novartis, personal fees from Daiichi-Sankyo, personal fees from MSD, personal fees from Kyowa Hakko Kirin, personal fees from Celgene, personal fees from Sumitomo Dainippon Pharma, grants and personal fees from Eisai, grants from REGENERON, grants from Astellas, grants from Gilead Sciences, grants from AMED (Japan Agency for Medical Research and Development), grants from JSPS (Japan Society for the Promotion of Science), personal fees from Ono Pharmaceutical Co., Ltd., and personal fees from Bristol-Myers Squibb.
